# Effects of the Healthy Children, Healthy Families, Healthy Communities Program for Obesity Prevention among Vulnerable Children: A Cluster-Randomized Controlled Trial

**DOI:** 10.3390/ijerph17082895

**Published:** 2020-04-22

**Authors:** Jina Choo, Hwa-Mi Yang, Sae-Young Jae, Hye-Jin Kim, Jihyun You, Juneyoung Lee

**Affiliations:** 1 Department of Community Health Nursing, College of Nursing, Korea University, Seoul 02841, Korea; ghkal@korea.ac.kr (H.-M.Y.); vital4@naver.com (H.-J.K.); jhemin87@naver.com (J.Y.); 2 Department of Sport Science, University of Seoul, Seoul 02504, Korea; syjae@uos.ac.kr; 3 Department of Biostatistics, College of Medicine, Korea University, Seoul 02841, Korea; jyleeuf@korea.ac.kr

**Keywords:** healthy lifestyle, childhood obesity, parenting, vulnerable populations

## Abstract

*Background*: We aimed to examine whether the Healthy Children, Healthy Families, and Healthy Communities Program, consisting of multi-level strategies for obesity prevention tailoring the context of socioeconomically vulnerable children based on an ecological perspective, would be effective on improving their healthy lifestyle behaviors and obesity status. *Methods*: Participants were 104 children (and 59 parents) enrolled in public welfare systems in Seoul, South Korea. Based on a cluster-randomized controlled trial (no. ISRCTN11347525), eight centers were randomly assigned to intervention (four centers, 49 children, 27 parents) versus control groups (four centers, 55 children, 32 parents). Multi-level interventions of child-, parent-, and center-level strategies were conducted for 12 weeks. Children’s healthy lifestyle behaviors and obesity status were assessed as daily recommended levels and body mass index ≥85th percentile, respectively. Parents’ parenting behaviors were measured by the Family Nutrition and Physical Activity scale. *Results*: Compared to the control group, the intervention group showed significant improvements in total composite scores of healthy-lifestyle behaviors—including 60-min of moderate physical activity—but not in obesity status among children. Moreover, the intervention group showed significant improvements in parenting behaviors among parents. *Conclusion*: The multi-level strategies for obesity prevention based on an ecological perspective may be effective for promoting healthy lifestyles among socioeconomically vulnerable children.

## 1. Introduction

The World Health Organization (2020) has identified childhood obesity as the most severe global health problem, in need of urgent resolution [1]. The severity of the problem may be more remarkable among socioeconomically vulnerable children, among whom the prevalence of overweight/obesity is almost twice that of their counterparts [2,3]. Particularly, childhood obesity is closely linked to adulthood obesity and cardiovascular risk [4,5]. Therefore, innovative strategies for preventing childhood obesity in vulnerable children are critical in protecting them from such latent health problems. 

In the present study, the Healthy Children, Healthy Families, Healthy Community Program (i.e., The Three-Healthy Program) was designed based on an ecological perspective to prevent obesity for socioeconomically vulnerable children in the public welfare system setting. An ecological perspective emphasizes the influences of multi-level environments on human behaviors, and has been effectively applied in the reduction of health inequalities [6,7]. In this context, we assume that multi-level interventions based on an ecological perspective, targeting children, parents, and organizations where children spend most of their time, may be strategies tailoring to the innate characteristics of socioeconomically vulnerable children. As a result, the intervention may maximize the promotion of healthy lifestyle behaviors and the prevention of childhood obesity. However, few studies have used this theoretical perspective systematically for childhood obesity prevention targeting vulnerable children. 

Healthy lifestyle behaviors, including healthy eating and physical activity, may be antecedent to healthy body weight, and must, therefore, be fostered during childhood. Healthy lifestyle behaviors such as the intake of fruits/vegetables [8] and non-/less sugar-sweetened beverages [9,10,11], increased physical activity [12], and reduced sedentary behaviors [9] are significantly associated with healthy body weight in children. Meanwhile, low rates of healthy lifestyle behaviors are more likely among socioeconomically vulnerable children. Such inequalities in healthy lifestyle behaviors may be attributable to their own poor family and organizational/community environments [13]. Inherent susceptibility [14], poverty [4], and body mass index (BMI) trajectories from childhood [15] have been studied in identifying obesity development and transition into adolescence and adulthood. Nonetheless, environments [16] should be paid attention, especially in addressing population-based health promotion measures from childhood obesity.

Based on an ecological perspective, human behavior may be viewed as occurring within multiple levels of influence. Bronfenbrenner (1979) first proposed an ecological perspective in child development [17], and McLeroy, Bibeau, Steckler, and Glanz (1988) modified it, focusing on health behaviors and, more practically, addressing the importance of ecological planning approaches [18]. McLeroy et al.’s (1988) ecological perspective may help community health professionals design effective tailored intervention strategies corresponding to every level (i.e., intrapersonal, interpersonal, organizational, and community/political) [18]. Particularly, children’s behaviors may be influenced by their surroundings, such as parents and organizations (schools or welfare systems) as well as their individual characteristics [19]. Therefore, parents and organizations (e.g., schools or welfare system settings) may be key targets in promoting children’s health behaviors. 

Parents play a vital role in promoting children’s healthy eating and activity behaviors and preventing obesogenic environments [20,21]. Good parenting behaviors may be correlated with improvements in children’s healthy lifestyle behaviors [22,23,24] and obesity status [25]. Therefore, cultivating obesity-specific parenting behaviors may be a primary target in the design of effective childhood obesity prevention interventions.

Organizational environments surrounding children should be considered as a modality for effective obesity prevention. Community centers designated for providing public welfare services are across countries (e.g., the Head Start Program in the US, Sure Start Program in the UK, and Community Child Center Program in South Korea) [26,27]. Particularly, the community child center in South Korea is an organization where children spend most of their after-school time [28]. Based on this background, organizations beyond the school could be used to effectively foster healthy lifestyle behaviors for socioeconomically vulnerable children. Furthermore, directors (or teachers) in the community child center setting could be sensible to identify not only which environment elements affect children’s health but also how they influence health behaviors and outcomes [29]. However, there is little evidence on the effects of organizational-level strategies on childhood prevention on a basis of public welfare system settings. 

In this context, the Three-Healthy Program involved parents and center directors as well as children themselves, applying multi-level strategies, that is, child-, parent-, and organization-level strategies tailoring the context of socioeconomically vulnerable children. The aim of the study was to examine the effects of the Three-Healthy Program on healthy lifestyle behaviors and obesity status in children in a public welfare system setting. Therefore, we hypothesized that an intervention group who received the Three-Healthy Program would have significant improvements in healthy lifestyle behaviors and significant reduction in obesity status, compared to a control group after 12 weeks.

## 2. Materials and Methods 

### 2.1. Study Design and Participants

**Figure 1:**
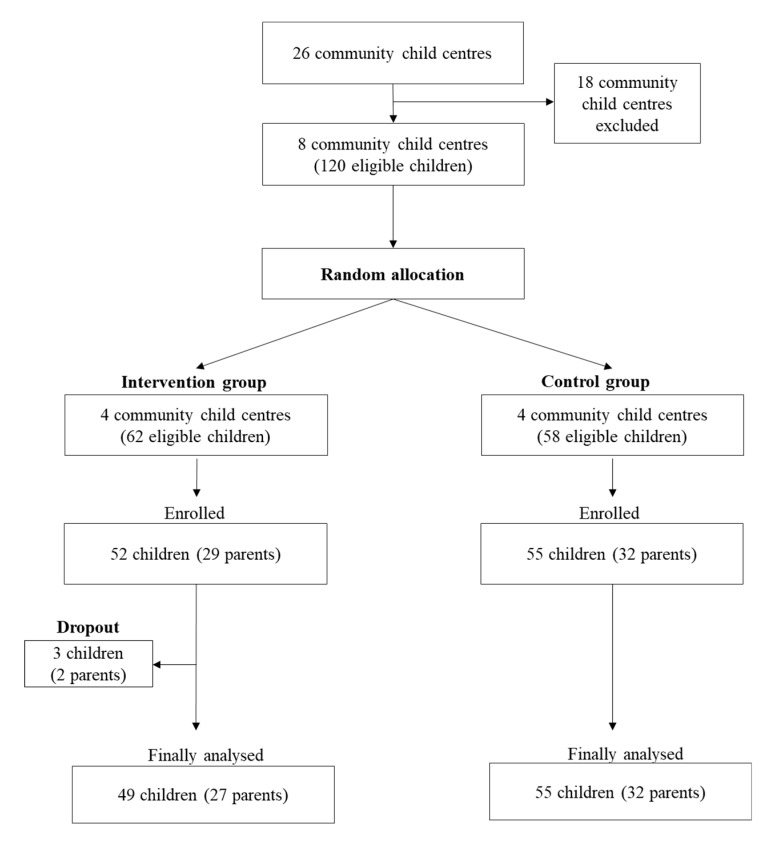
Participants’ flow chart.

This study was a cluster-randomized controlled trial, embedded in a larger parent study, ‘Development and Effects of the Healthy Children, Healthy Families, Healthy Communities Program (i.e., The Three-Healthy Program) for Obesity Prevention among Vulnerable Children: Using the Ecological Perspective’ (trial registration no. ISRCTN11347525), conducted from 2014 to 2017 [2,30,31]. The present study was conducted in community child centers of a public welfare setting; eight community child centers recruited for the present study were randomly allocated to an intervention group (four centers) and a control group (four centers). Participant recruitment was conducted from each of intervention and control groups (Figure 1) and the more details were as follows.

Participants were children (N = 104) and parents (N = 59), enrolled in eight community child centers in Seongbuk municipal county, Seoul. In the present study, socioeconomically vulnerable children were defined as those registered in the public welfare system of community child centers, which serve children from: (1) families receiving benefits from the National Basic Livelihood Security System and (2) non-traditional families including grandparent-grandchild and single-parent families [28].

Eligibility criteria for child participants were (1) in primary 3–6 grade schools and (2) no mental and physical disabilities. Eligible criteria for parent participants were (1) mothers, fathers, or legal representatives of the children who agreed to participate, (2) living with the children, and (3) no mental and physical disabilities. Of the child participants, 59 formed dyads with a parent. The unique characteristics of socioeconomically vulnerable families (grandparent-grandchild and single-parent families or parents’ busy schedules) made it difficult to recruit purely dyads [31].

Recruitment was conducted from June 12–28, 2017. The principal investigator contacted a steering group of 26 community child centers in Seongbuk county, and visited each one to explain the purpose and characteristics of the study. Eight centers agreed to participate, which had a total of 261 children, and then were randomly allocated to the intervention group (four centers) and the control group (four centers) (Figure 1). Of the 261 children, 121 children satisfied eligibility criteria for the study (i.e., 63 in the intervention group and 58 in the control group). Finally, of the 121 eligible children, 52 children (and 29 parents) of the intervention group and 55 children (and 32 parents) of the control group agreed to participate in the study. The intervention group received 12-week Three-Healthy Program, while the control group received usual care being provided in the community child center program. After enrolled in the study, three children and two parents in the intervention group were dropped out owing either to the children’s refusal to attend the educational sessions or their withdrawal from the community child center.

The minimum sample size of children was calculated according to the criteria for cluster-randomized trial design [32], because community child centers were allocated as clusters into intervention and control groups. We used BMI z-score as a primary outcome variable [33]. Chen, Kao, Hsu, Wang, and Hsu’s (2015) study showed a significant improvement in BMI z-scores among children, with means (standard deviations (SD)) of 2.8 (1.16) and 2.4 (1.09) in the intervention and control groups, respectively; the mean difference between two groups was 0.4 after a seven-week family-based intervention [33]. Accordingly, we took 0.8 as the mean difference based on the assumption that the present study may show a two times higher effect size than Chen et al.’s (2015) study because of the multi-level interventions in addition to the family-based intervention and the longer duration. By assuming an intracluster correlation coefficient among child’s BMI z-score within community child centers (ρ) as 0.01 and a number of centers per group (k) as 4, it is found that a total of 48 subjects per group (i.e., an average of 9 children for each community center per group) is needed to achieve 90% of power (1−β) to detect a mean difference of average BMI z-score change (d) of 0.8 with assuming its standard deviation (σ) of 1.13 under a two-sided significance level (α) of 0.05 [29]. This minimum required sample size of 96 children (48 per group) was satisfied in our study (N = 104). 

### 2.2. The Three-Healthy Programme

The 12-week intervention comprised ecological, multi-level intervention strategies: child-level educational strategies, parent-level strategies, and center-level organizational strategies for obesity prevention among vulnerable children (Table 1). 

The child-level intervention consisted of behavioral strategies based on the cognitive learning theory [34] such as goal setting, self-monitoring, reinforcement, problem-solving, and experiential learning activity strategies (e.g., cooking, taste, and exercise classes) [35] (Table 1). There were 12 weekly sessions of group teaching: six for healthy eating and six for healthy activity, respectively. The sessions targeted the achievement of the 5–2–1–0 lifestyle practice [36]: >5 times per day of fruit and vegetable intake, <2 h per day of sedentary behaviors, >1 h per day of exercise, and 0 per day sugar-sweetened beverage intake. The healthy eating sessions covered (1) the relation between healthy eating behaviors and health outcomes, (2) self-efficacy enhancement, and (3) the understanding of the Nutrition Facts label. The healthy activity sessions consisted of weekly exercise directed by physical education graduates, using flash cards to provide necessary information with (1) principles for desirable physical activity and health outcomes, (2) safe activity practices, and (3) disadvantages of sedentary behaviors. 

The parent-level intervention consisted of parenting strategies (Table 1): (1) promoting positive parenting styles and general/obesity-specific parenting practices and (2) building behavioral modification skills of goal setting, self-monitoring, and reinforcement, and fostering a supportive family environment. It involved one session of group teaching, two home visits, three telephone counselling sessions, and 12 weekly text messages. 

The center-level intervention consisted of organizational strategies such as partnership building, curriculum development, center staff education, and center policy changes (Table 1). Before initiating the intervention, the principal investigator from the university and all eight community child center directors signed a memorandum of understanding to build a partnership for participating in obesity prevention activities. The researchers developed a 12-session educational curriculum for healthy eating and activity classes and secured the physical environment (i.e., place and time) for operating educational classes after consulting with each center director. Moreover, the researchers educated the faculty members (directors, teachers, and cooks) to be aware of obesity prevention and its related positive behaviors, and motivated them to encourage children’s attendance in educational sessions and monitor their 5–2–1–0 lifestyle practices. Regarding policy changes, community child centers were encouraged to display posters regarding 5–2–1–0 lifestyle practices and to adopt policies such as no sugar-sweetened beverages and meals with daily provision of fruits and vegetables. Finally, collaborative activities with researchers, parents, and center directors, such as a workshop (to prevent barriers against healthy behaviors) and a walking festival (to foster sustainable children’s and parents’ activity behaviors), were included. 

### 2.3. Measures

#### 2.3.1. Participants’ Baseline General Characteristics

Children self-reported their general characteristics, including age, sex, primary elementary school grade, living status, and perceived socioeconomic status. Parents also self-reported them, including age, educational status, monthly household income, and health insurance; educational status was set as the mother/father’s highest degree. 

#### 2.3.2. Children’s Knowledge of Healthy Lifestyle Behaviors

Children reported their healthy lifestyle behavior knowledge through an 18-item questionnaire developed by the principal investigator based on inquiries regarding children’s awareness of the benefits and daily recommended levels for healthy eating and activities. The questionnaire specifically covered 10 items of the statements for healthy eating including breakfast, snaking and fast foods, reading nutrition labels, fruits and vegetables, and milk; and eight items for healthy activities including the durations and types of both physical activity and sedentary behaviors. Children responded to each item as ‘yes’ (correct response coded as 1) or ‘no’ (incorrect response coded as 0). Coded scores were summed (range 0–18). A higher score indicated a higher knowledge level. 

#### 2.3.3. Children’s Healthy Lifestyle Behaviors

Children’s eating behaviors were assessed by self-report of a single behavior, that is, breakfast, fruits, vegetable, milk, sugar-sweetened beverage, and fast food consumption, as well as family mealtimes, in response to the question, ‘How many times did you eat breakfast (or other single behavior) per week on average during the past three months?’ They were reported as none per week, one–two times per week, three–four times per week, five–six times per week, once per day, twice per day, or thrice or greater per day. The daily recommended levels of healthy eating behaviors were as follows: breakfast daily; fruit consumption ≥2 times per day; vegetable consumption ≥3 times per day; total milk consumption as either fluid milk or dairy products ≥1 time per day; sugar-sweetened beverages and fast food 0 time per day; family mealtime ≥1 time per day. 

Children’s activity behaviors were assessed by self-reports of physical activity and non-sedentary behavior. Physical activity was assessed as number of days per week with the question, ‘How many days a week did you exercise moderately or vigorously for 60 min on average during the past three months?’ Accordingly, days of sufficient physical activity (indicating moderate or vigorous exercise for 60 min per bout) were measured as a continuous variable (range 0–7). The recommended level of sufficient physical activity was defined as 60 min of moderate or vigorous activity seven days a week (Centers for Disease Control and Prevention, 2008) (satisfied = 1 vs. non-satisfied = 0). Non-sedentary behavior was assessed as number of hours per day spent on watching television, smartphone use, or computer games. The recommended level of non-sedentary behavior was <2 h per day [37].

A total composite score of nine healthy eating and activity behaviors for each child was as follows: each child received either a score of 1 or 0 for a single behavior (1 = meeting versus 0 = not meeting a recommended level of each health behavior). Individual scores were then summed to provide a composite score from 0–9.

#### 2.3.4. Children’s Obesity Status

Children’s obesity status was defined as ≥85th percentile of BMI calculated using body weight and height (kg/m2). According to the 2007 sex-specific BMI-for-age Korean National Growth Charts [38], children were categorized as normal weight (BMI < 85th percentile), overweight (85th percentile ≤ BMI < 95th percentile), or obese (BMI ≥ 95th percentile or BMI ≥ 25 kg/m^2^). Additionally, BMI z-scores were also measured, which indicate standardised BMI scores adjusted for child’s age and sex at a population level [39] and obtained from AnthroPlus [40]. Children’s body weight (kg) and height (cm) were measured with no shoes or outerwear using an electronic weight/body fat scale (HBF-212, Omron, Kyoto, Japan) and standing height scale (Seca 213, Seca GmbH & Co. KG., Hamburg, Germany), respectively. Prior to anthropometric measurement, children fasted for eight hours and emptied their bladders. 

#### 2.3.5. Parenting Behaviors

The Family Nutrition and Physical Activity (FNPA) scale [20] was used to assess parenting behaviors, specifically those related to obesity. The FNPA scale is a behavioral assessment designed to allow parents to evaluate obesogenic environments and practices that may predispose children to becoming overweight [41], consisting of 10 factors: family meal patterns, family eating habits, food choices, beverage choices, restriction/reward, screen time behavior and monitoring, healthy environment, family activity involvement, child activity involvement, and family routine. It contains 20 items (two items per factor) measured on a four-point Likert scale (1 = almost never, 2 = sometimes, 3 = usually, and 4 = almost always; possible total scores range from 20–80). There are six negatively worded items (3, 4, 5, 7, 10, and 13), which are reversely scored. A higher FNPA score implies a higher level of obesity-specific parenting behavior. 

### 2.4. Ethical Considerations

All participants provided written informed consent after receiving an explanation of the study purposes. Children gave consent to participate, and parents provided consent for their own participation or that of their children. The study was approved by the concerned Institutional Review Board (No. 1040548-KU-IRB-17-82-A-2).

### 2.5. Data Analysis

Study subjects’ baseline characteristics were summarized as mean (standard deviation) for continuous variables and number (percentage) for categorical variables. Independent t-test or chi-square test was used to compare subject’s demographic variables between intervention and control groups. To test a homogeneity of baseline variables between intervention and control groups, and to identify significant changes from baseline (pre-test) to follow-up (post-test) measurements within group, univariable generalized estimating equations (GEE) were used to account for a clustering (community child centers) effects. An identity link function with the normal distribution for continuous outcome variables and a logit link function with binomial distribution for dichotomous outcome variables were used. Baseline imbalance (*p* < 0.05) between groups were identified in variables of the total composite score for healthy lifestyle behaviors, fruits, vegetables, milk consumptions and non-sedentary behavior. Therefore, univariable and multivariable GEE’s without and with adjusting the variables shown baseline imbalance, respectively, were used to compare post-test effects on outcome variables between groups, after adjusting pre-test measurements. In all of GEE analysis, an exchangeable variance-covariance structure within cluster was employed. All statistical analyses were performed using the SAS software, version 9.4 (SAS Institute, Cary, NC). All reported *p*-values were two-sided, and we considered a *p*-value < 0.05 to be significant. 

## 3. Results

The children had a mean age of 10.0 years (range: 8–12 years) and 45.2% were girls (Table 2). Of the children, 21.2% lived without their parents, and 57.7% perceived their socioeconomic status as low. The parents had a mean age of 44.0 years (range: 27–62 years); 57.6% had an educational level lower than college; 33.9% had a monthly income of less than $1754, positioning them at South Korea’s poverty threshold [42]; and 13.6% were Medicaid beneficiaries. There were no significant differences in children’s and parents’ baseline general characteristics (Table 2). 

With regard to the intervention effects (Table 3), children in the intervention group showed significant improvements in knowledge of healthy lifestyle behaviors from pre-test (mean (SD) = 13.0 (1.74)) to post-test (mean (SD) = 14.8 (1.45)), compared to those in the control group (*p* = 0.026) (Table 3). Children in the intervention group showed significant improvements in total composite scores of healthy lifestyle behaviors from pre-test (mean (SD) = 2.4 (1.56)) to post-test (mean (SD) = 3.4 (2.12)) compared to the control group (*p* < 0.001); recommended vegetable intake from pre-test (n (%) = 5 (10.2)) to post-test (n (%) = 13 (26.5)) compared to the control group (*p* = 0.011); and recommended family meal time from pre-test (n (%) = 22 (44.9)) to post-test (n (%) = 23 (46.9)) compared to the control group (*p* = 0.001). However, children in the intervention group did not show any significant improvements in breakfast, fruit, milk, no-sugar-sweetened beverage, and fast food consumption from pre- to post-test, compared to those in the control group. 

For healthy activity behaviors, children in the intervention group showed significant improvements in days of sufficient physical activity from pre-test (mean (SD) = 2.8 (2.25)) to post-test (mean (SD) = 4.3 (2.16)) compared to those in the control group (*p* = 0.001) (Table 3). Moreover, sufficient physical activity from pre-test (n (%) = 6 (12.2)) to post-test (n (%) = 13 (26.5)) compared to the control group (*p* = 0.043). However, the intervention group did not show any significant improvements in non-sedentary behaviors compared to the control group. For obesity status and BMI z-scores, there were no significant differences between the groups. 

Finally, regarding the intervention effect on parenting behaviors, parents in the intervention group showed significant improvements in FNPA scores from pre-test (mean (SD) = 49.1 (7.90)) to post-test (mean (SD) = 54.9 (9.12)) compared to those in the control group (*p* < 0.001).

## 4. Discussion

The Three-Healthy Program, comprising multi-level strategies tailored for socioeconomically vulnerable children in public welfare systems, was associated with significant improvements in total composite scores of healthy-lifestyle behaviors among children and obesity-specific parenting behaviors among parents. However, it did not reveal a significant effect on children’s obesity. 

Children in the intervention group, compared to those in the control group, showed significant improvements in knowledge levels and total composite scores of healthy-lifestyle behaviors, specifically single behaviors such as vegetable consumption, family mealtimes, and days of sufficient activity. Few intervention studies have broadly covered multi-component healthy lifestyle behaviors as outcome measures in the context of childhood obesity prevention. Most studies have explored effects mostly on physical activity and fruit/vegetable consumption in school-based settings. Similar to our study, Nystrom et al. (2017) reported that a six-month mobile-based intervention to reduce obesity targeting 315 Swedish children aged 4.5 years was associated with a significant increase in total composite scores of healthy lifestyle behaviors (no significant effects on any single behavior) [43]. However, they did not find any significant decrease in obesity status as measured by fat mass index. Gorely, Nevill, Morris, Stensel, and Nevill (2009) did not assess the composite measure, but reported that a 10-month school-based intervention targeting 589 primary school children aged 7–11 showed significant effects on total time and daily steps in physical activity, but no significant effects on knowledge levels and fruit/vegetable consumption [44]. Ardic and Erdogan (2017) reported that a 15-week school-based intervention targeting 88 adolescents showed significant effects on daily steps and fruit/vegetable consumption during a 12-month follow-up [45]. Based on this review, we may conclude that it is not possible to concretely compare our findings with previous results because of the high data heterogeneity across previous intervention studies in terms of populations, settings, intervention characteristics (duration, intensity, or contents), and outcome measurements. 

Nevertheless, in the present study, the significant effects of the Three-Healthy Program on total composite scores of healthy lifestyle behaviors may be partly attributable to the 12 sessions of the educational curriculum including ‘first-hand experience classes’: buzz sessions for writing and presenting to share individual experiences of healthy eating and activities, group cooking and exercise sessions. Furthermore, self-efficacy enhancement strategies for children and parents may be partly associated with increased total composite scores of healthy-lifestyle behaviors. They included persuasion through facilitating outcome expectancy and setting behavioral goals, frequent performance feedback of self-monitoring, problem solving with perceiving and overcoming barriers, and role modeling with demonstration of cooking and exercise [46]. Self-efficacy enhancement may promote healthy lifestyle behaviors among adults [47,48]. Nezami et al. (2016) reported that increases in eating self-efficacy and physical self-efficacy during the first half period of a 12-month intervention were predictive of healthy eating and activity behaviors at 12 months among 363 middle-aged adults [48]. However, few studies reported a link of self-efficacy enhancement to behavioral outcomes among children. Kulik et al. (2019) reported that eating self-efficacy significantly predicted healthy eating behaviors among children with a mean age of 9.9 years [49]. Meanwhile, in the present study, an increase in self-efficacy over a short duration of three months might be supposed, although it was not measured. Future studies are needed to reveal changes in self-efficacy over the intervention and their effects on healthy lifestyle behaviors among children. 

Parents were directed to monitor children’s five behaviors in a paper diary (fruit consumption, vegetable consumption, sedentary TV watching, physical activity, no sugar-sweetened beverage consumption), to be sent to the centers. Center directors were also directed to assess parents’ adherence to writing and submitting the paper diaries and to develop meal and snack service policies including fruit/vegetables and no-sugar-sweetened beverages. The researchers periodically provided feedback and reinforcements on behavioral adherence success and failure to center directors and parents, and arranged a workshop to resolve barriers to non-adherence. These interactive activities helping key stakeholders monitor children’s health behaviors and solve problems may be a crucial factor in our intervention’s positive effects on promoting healthy lifestyle behaviors. More importantly, center directors in the intervention group were actively committed to formulating new policies regarding meal services and curriculum changes, which may have enhanced the effects of interactive activities. Such implicit stakeholder activities embedded in multi-level strategies for childhood obesity prevention, which are not objectively measured, may have led to changes in healthy lifestyle behaviors of socioeconomically vulnerable children.

We found that parents in the intervention group, compared to those in the control group, showed significant improvements in FNPA scores of obesity-specific parenting behaviors. Parental involvement has been emphasized as a critical intervention strategy in childhood obesity prevention, particularly in school-based settings [50]; however, to the best of our knowledge, few studies have included an intervention for promoting parenting behaviors or even evaluated parental involvement levels as an outcome variable.

On the contrary, several studies have evaluated them in childhood obesity management (or weight management) in home-based or primary care settings. Tucker, DeFrang, Orth, Wakefield, and Howard (2019) reported that a six-month parent-based weight management programme showed a significant effect on improving FNPA scores, but not on children’s BMI in primary care settings [51]. Reportedly, higher FNPA scores were positively associated with children’s healthy eating behaviors and negatively associated with children’s obesity status [52,53]. This association was not confirmed in the present study (data not found). However, it may be reasonable to assume that the Three-Healthy Program’s effects on FNPA scores may have latent impacts on promoting healthy lifestyle behaviors and preventing obesity among children if good parenting behaviors are sustained for a long-term period. Our study was not able to involve all eligible parents owing to their busy work lives and the absence of parents in socioeconomically vulnerable families [31]. This lack of parental participation might have lowered the magnitude of intervention effects on obesity status.

We found that obesity status (as measured by either prevalence of obesity or BMI z-score) was not significantly reduced in the intervention group compared to the control group. Oosterhoff et al. (2016) conducted a meta-analysis on the effects of school-based lifestyle interventions and reported that a total of 85 randomized control trials revealed significant pooled effects of interventions on the reduction in children’s BMI [50]. Unlike this finding, such a non-significance in the reduction in obesity status may be partly explained by the relatively short duration of the intervention. According to Verjans-Janssen’s (2018) systematic review, at least one year may be needed to improve obesity status [54]. However, Bhave et al. (2016) reported no significant effects of a five-year school-based intervention on obesity status among 865 Indian children aged 7–10 [55]. Similarly, Lloyd et al. (2018) reported no significant effects of a school-based intervention on preventing overweight and obesity after 24 months among 1244 English children aged 9–10 [56].

Reviewing the above two studies, our finding may be also explained by intervention goals targeting specific lifestyle behaviors embedded in a certain population group. Exploring barriers to and facilitators of obesity-related behaviors in a specific study population should be prioritized before conducting a lifestyle intervention. Particularly, socioeconomically vulnerable children may face certain barriers to healthy eating behaviors, such as emotional eating resulting from fulfilling deprivation [31,57]. Moreover, Geserick et al. (2018) reported that approximately half of obese adolescents (aged 15–18 years) had been overweight or obese from 5 years of age and the trajectory of obesity prevalence showed a gradually increasing trend over the period of 0–14 years of age [15]. Of the participants in our study, 35.6% were overweight/obese children who were school-aged at 8–12 years. Based on Geserick’s finding, our participants may have been in the trajectory of increasing obesity and such trajectory might outweigh the intervention effects in our study. In this context, we suggest implementing more intensive strategies customized to population-specific causes of obesity-related behaviors and applying an early intervention at preschooler age.

Our study has several limitations. First, the non-randomized trial might have been related to the between-group differences in baseline measures, even though we used a random allocation of community child centers as clusters. They may have led to potential biased results. However, we adjusted the baseline measures in the statistical models to test main effects, which may minimize the potential bias. Second, children’s healthy lifestyle behaviors were self-reported, not collected through more objective measures such as a food frequency questionnaire (or diet recall diary) for eating behaviors and pedometer for physical activity. This may have resulted in overestimated or underestimated outcomes. Third, all participants were not children-parent dyads owing to the features of socioeconomically vulnerable families as mentioned above. This might have led to an underestimation of intervention effects. Finally, since the sample was limited to a vulnerable population, the results cannot be generalized to general populations. Authors should discuss the results and how they can be interpreted in perspective of previous studies and of the working hypotheses. The findings and their implications should be discussed in the broadest context possible. Future research directions may also be highlighted.

## 5. Conclusions

In conclusion, the Three-Healthy Program with multi-level strategies based on an ecological perspective may be effective for promoting healthy lifestyles among socioeconomically vulnerable children in public welfare systems, as well as parenting behaviors. More intensive strategies targeting population-specific obesity-related behaviors and greater parental involvement could lead to a reduction in childhood obesity.

## Figures and Tables

**Table 1:** The Three Healthy Program: multi-level intervention strategies.

Level	Strategies	Contents
**Child-level**	Goal setting Self-monitoring Reinforcement Problem-solving Experiential learning activities	Six sessions of group teaching for healthy eating - Principles of 5–2–1–0 lifestyle practices - Healthy eating behaviors and health effects; understanding of Nutrition Fact label - Self-efficacy enhancement for consuming fruits/vegetables and restricting sugar-sweetened beverages Six sessions of group teaching for healthy activity - Principles of desirable physical activity and health benefits - Safe physical activity practices; disadvantages of sedentary behaviors
**Parent-level**	Goal setting Parent-led monitoring Reinforcement Supportive family environment	One group teaching session - Principles of 5–2–1–0 lifestyle practices - Principles of general/specific parenting for obesity prevention Two home visits - Counselling for customized parenting practices - Adjusting the home environment Three telephone counselling sessions - Monitoring parenting practices - Enhancing parents’ self-efficacy in parenting Twelve text messages
**Center-level**	Building a partnership Organizing educational curriculum Educating faculty Policy changes Collaborative activities	Building a partnership with university and centers Organizing educational curriculum - Building physical environment for operating educational curriculum: designating a room and time Educating center directors and cooks - Encouraging children’s attendance in group sessions - Monitoring children’s behaviors Center policy changes - Supporting 5–2–1–0 lifestyle practices (including putting on a poster for 5–2–1–0 lifestyle practices) - Setting no sugar-sweetened beverage policy - Setting a meal serving policy (i.e., daily provision of fruits and vegetables) Collaborative activities with researchers, parents, and directors - Holding a workshop to prevent barriers against healthy behaviors - Holding a walking festival

Center indicates community child center.

**Table 2:** Participants’ baseline general characteristics.

Characteristics	n (%) or Mean (SD)						t/χ^2^	*p*
	Total		Intervention Group		Control Group			
**Children**								
No. of children (%)	104	(100)	49	(47.1)	55	(52.9)		
Age (years)	10.0	(1.23)	9.9	(1.18)	10.1	(1.27)	0.96	0.341
Sex							<0.01	0.955
Girl	47	(45.2)	22	(44.9)	25	(45.5)		
Boy	57	(54.8)	27	(55.1)	30	(54.5)		
Living with parents							0.09	0.760
Yes	82	(78.8)	38	(77.6)	44	(80.0)		
No	22	(21.2)	11	(22.4)	11	(20.0)		
Perceived SES							1.69	0.194
High	44	(42.3)	24	(49.0)	20	(36.4)		
Low	60	(57.7)	25	(51.0)	35	(63.6)		
Obesity status							0.35	0.557
Obese (BMI ≥ 85 %tile)	37	(35.6)	16	(32.7)	21	(38.2)		
Normal (BMI < 85 %tile)	67	(64.4)	33	(67.4)	334	(61.8)		
BMI z-score	1.1	(1.31)	0.8	(1.36)	1.3	(1.24)	1.83	0.070
**Parents**								
No. of parents (%)	59	(100)	27	(45.8)	32	(54.2)		
Age (years)	44.0	(5.51)	42.8	(5.67)	44.9	(5.26)	1.52	0.135
Educational status							3.31	0.069
≤College	25	(42.4)	8	(29.6)	17	(53.1)		
< College	34	(57.6)	19	(70.4)	15	(46.9)		
Monthly income ($)							1.04	0.308
High (> 1,754)	39	(66.1)	16	(59.3)	23	(71.9)		
Low (< 1,754)	20	(33.9)	11	(40.7)	9	(28.1)		
Health insurance							1.05	0.307
Medicare	51	(86.4)	22	(81.5)	29	(90.6)		
Medicaid	8	(13.6)	5	(18.5)	3	(9.4)		

BMI = body mass index; SD = standard deviation; SES = socioeconomic status.

**Table 3:** Effects of the Three Healthy Program for socioeconomically vulnerable children and their parents.

Outcome Variables	Intervention Group					Control Group					*p* ‡	*p* §
	Pre-Test		Post-Test		z	Pre-Test		Post-Test		z		
	N (%) or Mean (SD)					N (%) or Mean (SD)						
**Children (n = 104)**												
Knowledge levels	13.0	(1.74)	14.8	(1.46)	5.04 ^†^	13.7	(2.04)	14.2	(1.88)	3.01 ^†^	0.044	0.026
**Healthy behaviors**												
Total composite score	2.4	(1.56) ^*^	3.4	(2.12)	21.84 ^†^	3.3	(1.68)	2.9	(1.62)	−1.25	0.178	<0.001
Healthy eating behaviors												
Breakfast (yes, daily)	25	(51.0)	30	(61.2)	3.08 ^†^	33	(60.0)	31	(56.4)	−2.20 ^†^	0.562	0.128
Fruits (≤2 times/day)	5	(10.2) ^*^	12	(24.5)	2.70 ^†^	15	(27.3)	14	(25.5)	−0.34	0.940	0.376
Vegetables (≤3 times/day)	5	(10.2) ^*^	13	(26.5)	2.14 ^†^	17	(30.9)	9	(16.4)	−2.79 ^†^	0.241	0.011
Milk (≤1 time/day)	24	(49.0) ^*^	29	(59.2)	1.05	41	(74.6)	32	(58.2)	−3.50 ^†^	0.925	0.634
Non-SSB (0 time/day)	6	(12.2)	8	(16.3)	1.15	9	(16.4)	9	(16.4)	0.0	0.999	0.766
Fast food (0 time/day)	8	(16.3)	21	(42.9)	2.41 ^†^	13	(23.6)	18	(32.7)	1.12	0.303	0.628
Family meal (≤ 1/day)	22	(44.9)	23	(46.9)	0.57	18	(32.7)	14	(25.5)	−1.44	0.010	0.001
Healthy activity behaviors												
Days of sufficient PA	2.8	(2.25)	4.3	(2.16)	2.51 ^†^	3.8	(2.01)	3.4	(2.29)	−1.04	0.014	0.001
Sufficient PA (7 days/wk)	6	(12.2)	13	(26.5)	1.99 ^†^	7	(12.7)	8	(14.6)	0.25	0.206	0.043
Non-sedentary behavior	15	(30.6) ^*^	17	(34.7)	0.42	29	(52.7)	26	(47.3)	−1.23	0.038	0.438
**Obesity status**	16	(32.7)	18	(36.7)	2.50 ^†^	21	(38.2)	23	(41.8)	2.62 ^†^	0.689	0.490
**BMI z-score**	0.8	(1.36)	0.9	(1.36)	2.71 ^†^	1.3	(1.24)	1.3	(1.22)	0.30	0.173	0.050
**Parents (n = 59)**												
FNPA score	49.1	(7.90)	54.9	(9.12)	−4.13 ^†^	49.4	(7.24)	49.4	(7.40)	0.0	<0.001	<0.001

BMI = body mass index; FNPA = Family Nutrition and Physical Activity; PA = physical activity; SD = standard deviation; SSB = sugar-sweetened beverages. * Significant between-group difference at baseline (pre-test) by method of generalized estimating equation (GEE) using an identity link function with normal distribution for quantitative variables or using a logit link function with binomial distribution for dichotomous variables. ^†^ Significant within-group changes from pre-test to post-test by GEE. ^‡^ *p*-value by univariable analysis for between-group post-test difference after adjusting pre-test with GEE. ^§^ *p*-value by multivariate analysis for between-group post-test difference after adjusting pre-test and variables shown pre-test imbalance between groups (i.e., Total composite score, Healthy eating behaviors of Fruits, Vegetables, Milk, and Non-sedentary behavior) with GEE. An exchangeable variance-covariance structure within cluster (community child center) was assumed in all GEE analyses.
